# A phase 1b, multicentre, dose escalation, safety and pharmacokinetics study of tilvestamab (BGB149) in relapsed, platinum-resistant, high-grade serous ovarian cancer (PROC) patients

**DOI:** 10.1038/s41416-025-03090-6

**Published:** 2025-07-22

**Authors:** Kenneth Sooi, Tuan Zea Tan, Jae-Weon Kim, Jung Yun Lee, Byoung-Gie Kim, David Micklem, Akil Jackson, David J. Pinato, Charlie Gourley, Rebecca Kristeleit, Sarah P. Blagden, Line Bjorge, David Shao Peng Tan

**Affiliations:** 1 National University Hospital, Singapore, Singapore; 2 National University Cancer Institute, Singapore, Singapore; 3 Cancer Science Institute of Singapore, Singapore, Singapore; 4 Genomics and Data Analytics Core, Singapore, Singapore; 5 Seoul National University, Obstetrics and Gynecology, Seoul, Republic of Korea; 6 Yonsei Cancer Center, Severance Hospital, Yonsei University College of Medicine, Seoul, Republic of Korea; 7 Samsung Medical Center, Sungkyunkwan University School of Medicine, Seoul, Republic of Korea; 8 BerGenBio ASA, Research, Bergen, Norway; 9 BerGenBio Ltd, Clinical Development, Oxford, UK; 10 Imperial College London, Hammersmith Hospital, London, UK; 11 University of Piemonte Orientale, Division of Oncology, Department of Translational Medicine (DIMET), Novara, Italy; 12 Western General Hospital, Edinburgh Cancer Centre, Edinburgh, UK; 13 Cancer Research UK Scotland Centre, Institute of Genetics and Cancer, University of Edinburgh, Edinburgh, UK; 14 Department of Oncology, Guys and St Thomas’ NHS Foundation Trust, London, UK; 15 Comprehensive Cancer Centre, King’s College London, London, UK; 16 University of Oxford, Department of Oncology, Churchill Hospital, Oxford, UK; 17 Oxford University NHS Foundation Trust, Early Phase Clinical Trials Unit, Oxford, UK; 18 University of Bergen, Haukeland University Hospital, Bergen, Norway; 19 University of Bergen, Department of Clinical Science, Center for Cancer Biomarkers CCBIO, Bergen, Norway; 20 National University Centre for Cancer Research (N2CR), Yong Loo Lin School of Medicine, National University of Singapore, Singapore, Singapore

**Keywords:** Ovarian cancer, Predictive markers, Clinical pharmacology

## Abstract

**Background:**

Tilvestamab is a highly selective humanised immunoglobulin G1 anti-AXL monoclonal antibody. This phase 1 study evaluated its optimal dose, safety, tolerability, immunogenicity and pharmacokinetics (PK) in relapsed platinum-resistant HGSOC patients.

**Methods:**

Patients received tilvestamab in three dose levels (1 mg/kg, 3 mg/kg and 5 mg/kg) via IV infusion every 2 weeks. Primary objectives included safety, tolerability and PK. Exploratory objectives included overall response, progression-free survival (PFS) and quality-of-life measures. Pharmacodynamic included AXL expression, gene and protein changes by transcriptomic and proteomic analysis.

**Results:**

Between 25 February 2021 and 4 February 2022, 16 patients were enroled across 8 sites in Singapore, Korea, United Kingdom, and Norway. Median treatment duration was 6.1 weeks. Grade 3 or higher treatment-emergent adverse events occurred in 62.5% patients, but none were tilvestamab-related. Common events included fatigue (38%), anorexia (38%) infections (31%), anaemia (25%) and dyspnoea (25%). No objective responses were observed, but 7 (44%) had stable disease at 6 weeks. PK showed dose-proportional exposure and steady-state by the second dose. Pharmacodynamic analyses revealed reduced fibrosis-related gene signatures and AXL protein expression. Epithelial-mesenchymal transition reversal was seen in 2 patients.

**Conclusion:**

Tilvestamab was well-tolerated and further studies to examine the efficacy of AXL inhibition in other indications are required.

**Clinical trial registration:**

This trial is registered at https://clinicaltrials.gov. Registration number: NCT04893551. EudraCT Number: 2020-001382-36

## Introduction

Epithelial ovarian cancer (EOC) is the most lethal gynaecologic malignancy, accounting for approximately 90% of all ovarian cancer cases [1, 2], with high-grade serous ovarian cancer (HGSOC) being the most common subtype [3]. Patients with advanced HGSOC are primarily treated with debulking surgery and platinum-based chemotherapy. Unfortunately, most patients develop recurrent disease. Patients who experience disease relapse within 6 months of completing platinum-based chemotherapy define platinum-resistant ovarian cancer (PROC) [4, 5]. Patients with PROC have limited therapeutic options. Current consensus guidelines from the European Society for Medical Oncology and the European Society of Gynaecological Oncology agree that there is no validated clinical biomarker available to determine how likely a patient is to develop platinum resistance in clinical practice [6]. As such, there is a need for continued efforts to find more effective treatments for PROC.

AXL is a transmembrane protein kinase belonging to the TYRO3-AXL-MERTK (TAM) family of receptors [7], essential for maintaining balance of the immune, haematopoietic and vascular systems [8]. Aberrant TAM receptor signalling is often associated with fibrosis and autoimmune disorders [8, 9]. AXL is expressed in many cancer types and has been associated with poor clinical prognosis and outcomes [10–13]. Rather than functioning as an oncogenic driver, AXL and its ligand growth arrest specific 6 (GAS6) protein are implicated in important components of the metastatic cascade, including promotion of tumour cell proliferation, survival, migration, invasion, angiogenesis, immune evasion and epithelial-to-mesenchymal transition (EMT) [14, 15]. Additionally, AXL/TAM receptors play important roles during tissue modelling in fibrotic diseases. The same GAS6-TAM receptor pathway that transforms epithelial-like cancer cells into mesenchymal cells seem to be adopted by tissues under chronic inflammation to form fibrotic scars [16], and multiple pre-clinical and murine models have shown that targeting of TAM receptors help to reduce fibrosis and inflammation in idiopathic pulmonary fibrosis, kidney fibrosis and liver fibrosis [17–21]. While AXL-associated EMT promotes resistance to targeted therapy, it can be inhibited. Inhibition of AXL has been shown to block tumour formation, metastasis, and reverse drug resistance in several experimental cancer models, including ovarian cancer [22–24]. Responses to the AXL inhibition have been correlated with protein expression (via IHC) or gene expression (via microarray analysis) in preclinical and animal studies [22, 24, 25].

In metastatic ovarian serous adenocarcinoma, AXL overexpression by immunohistochemistry (IHC) has been demonstrated in 88% of primary tumours, 75% of omental, and 90% of peritoneal deposits. AXL is not expressed in normal ovarian epithelium [25]. Silencing AXL or blocking the GAS6-AXL pathway has been shown to prevent regional dissemination of ovarian cancer cells in vivo, demonstrating AXL to be a critical factor in ovarian tumour metastasis [25].

At least five distinct gene expression molecular subtype (GEMS) of EOC have been identified: epithelial-A/differentiated/C3, epithelial-B/immunoreactive/C2,C4, mesenchymal/C1, stem-A/proliferative/C5 and stem-B/C6 [26–28]. Pre-clinical data suggests that AXL is a mesenchymal subtype specific therapeutic target in HGSOC [22, 23]. An AXL-related gene expression signature pattern correlated with cellular plasticity and transition from epithelial towards mesenchymal subtypes, and was associated with resistance to chemotherapy and targeted therapy. In mesenchymal subtype ovarian cancer cell lines, crosstalk between GAS6 activation of AXL and receptor tyrosine kinases (HER2 and MET) and its downstream signalling served specifically to enable cell mobility in mesenchymal cells, a feature absent from other molecular subtypes. The dependence of mesenchymal cell lines on the GAS6-AXL axis renders them sensitive to pharmacologic inhibition of AXL, and activity has been shown with the small molecule AXL kinase inhibitor bemcentinib across various cancer types including non-small cell lung cancer, mesothelioma and acute myeloid leukaemia [29–32].

Tilvestamab is a highly selective full-length humanised immunoglobulin G1 anti-AXL monoclonal antibody. By inhibiting AXL kinase, tilvestamab is expected to modulate key fibrogenic pathways such as epithelial/endothelial to mesenchymal transition [33] and macrophage function and transforming growth factor β transcription [34, 35]. In addition, tilvestamab is expected to directly inhibit the deposition of extracellular matrix by myofibroblasts [19]. Tilvestamab may therefore have the potential to prevent cancer cell survival mechanisms and limit disease progression in patients with relapsed, platinum-resistant HGSOC [36]. A single ascending dose study in healthy male volunteers, who were administered a single dose of tilvestamab at one of 4 dose levels (0.1 mg/kg to 3.0 mg/kg) demonstrated that tilvestamab was generally well tolerated at all doses with biochemical, haematologic or immunological toxicities being observed [37]. Further details can be found in the supplementary index **(**supplementary material 1**)**.

In this phase 1 study, we aimed to evaluate the optimal dose of tilvestamab, as well as its safety, tolerability and pharmacokinetics in patients with platinum-resistant HGSOC.

## Methods

### Study design and participants

This was an open-label, multicentre, prospective, single-arm, dose escalation phase 1 study to assess the safety, pharmacokinetics (PK) of tilvestamab given as a monotherapy to adults with relapsed platinum-resistant HGSOC (Clinicaltrials.gov identifier NCT04893551). The trial was conducted in accordance with protocol requirements, the International Conference on Harmonisation for Good Clinical Practice and the guiding principles in the Declaration of Helsinki, according to local laws and regulations in each participating country. All enroled patients provided written informed consent before undergoing study-specific procedures.

Eligible patients were recruited from 8 sites in Singapore, South Korea, Norway, UK and Germany. Inclusion criteria required all patients to be females of non-childbearing potential at least 18 years of age; with histologically documented HGSOC that have experienced a platinum-resistant relapse, defined as progressive disease on imaging within ≤6 months from completion of most recent platinum-based regimen; received no more than 4 prior lines of systemic therapy; an Eastern Cooperative oncology Group performance status of 0 or 1; and adequate bone marrow, liver, kidney, coagulation and cardiac function. Exclusion criteria included radiation therapy or anticancer therapy or investigational medicinal product within 28 days of enrolment, active brain metastases, unstable cardiovascular function, inflammatory bowel disease, uncontrolled active infection (Hepatitis B and C infection were not excluded), HIV infection, concurrent malignancies (except non-melanoma skin cancer or carcinoma in-situ of the cervix) and use of warfarin or warfarin derivatives (treatment with low molecular weight heparin permitted).

### Study schedule

This study evaluated escalating doses of tilvestamab at 1 mg/kg, 3 mg/kg and 5 mg/kg given by intravenous infusion every 2 weeks. Each treatment cycle spanned over 28 days. The total accrual target was 24 patients.

In the dose escalation phase, patients were assigned to receive tilvestamab at a dose level of 1 mg/kg (cohort A), 3 mg/kg (cohort B) or 5 mg/kg (cohort C). In each cohort, the first patient to receive tilvestamab infusion was considered the sentinel patient. If no safety concerns were identified for up to 48 h, an additional 3 patients were enroled into the cohort. After a total of 4 patients had been enroled at each dose level (starting with cohort A), the Protocol Steering Committee (PSC) reviewed the cycle 1 safety and PK data for all patients in the ongoing cohort before deciding on escalation to subsequent cohorts (cohorts B and C).

**Fig. 1:**
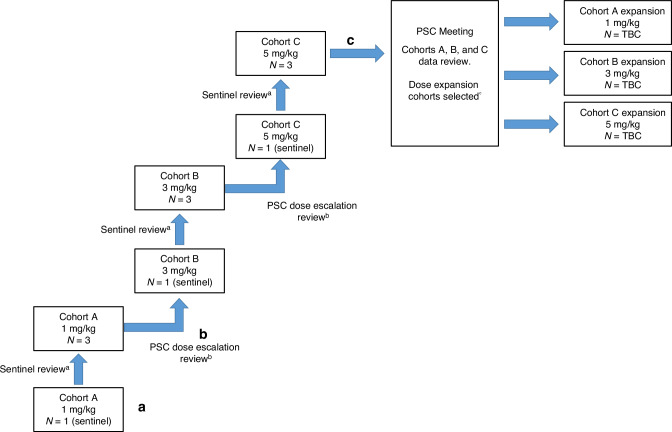
Study Design. Flowchart illustrating patient recruitment at various timepoints. N number of patients, PSC Protocol Steering Committee, TBC to be confirmed. **a** The first enrolled patient to receive tilvestamab infusion at each dose level was considered to be a sentinel patient, in whom administration was conducted in isolation, with no further enrolment at that dose level until review of safety information collected up to 48 hours after first dose administration. Review was performed by the Sponsor’s chief medical officer or medical monitor and the principal investigator. **b** The PSC review took place after all patients had completed Cycle 1. **c** The PSC reviewed all available safety, pharmacokinetic, and pharmacodynamic data to decide which cohorts should be reopened for enrolment of a further 12 patients overall.

In the dose expansion phase, after the 3 cohorts had each enrolled 4 patients, the PSC reviewed all safety and PK data obtained from patients’ first cycle of tilvestamab administration. The PSC then determined whether to expand any or all the dose cohorts for enrolment of up to 12 additional patients. Figure 1 shows a schematic representation of the study design.

Tilvestamab treatment was continued until tumour progression, death, unacceptable toxicity, or withdrawal of consent. Pertaining to safety, dose escalation would be stopped and further dosing at the same or higher dose levels would not be initiated if dose limiting toxicities (DLT) criteria were met. DLT criteria were as follows: clinically significant adverse events (AEs) occurring in two or more patients that in the opinion of the PI warrant cessation of dose escalation, two or more patients experiencing grade 3 or higher AEs, one or more patients experiencing a serious AE, one or more patients having an increase of more than 60 ms in QTcF compared with baseline value or have a QTcF of >500 ms. With respect to PK, dose escalation would be stopped if PK data indicated that the predefined maximum clinical exposure level had been achieved, or was predicted to be achieved at the next dose level, or if the PK data appeared to be unpredictable.

### Outcomes

The primary objective was to assess the safety, tolerability and PK of single-agent tilvestamab. Plasma PK exposure was determined by comprehensive profiling (at single dose and steady-state) of multiple ascending doses of tilvestamab. The secondary objective was to assess the immunogenicity of tilvestamab. Exploratory objectives included determination of overall response rate (ORR), disease control rate (DCR), progression-free survival (PFS) and quality-of-life (QOL) measures in the overall population as well as AXL IHC-positive patients. Additional objectives included determination of qualitative and quantitative pharmacodynamic effects of tilvestamab, assessment of relevant biomarkers in tumour and blood that support immune modulation and AXL signalling.

### Safety assessment

All AE were recorded and graded using the National Cancer Institute Common Terminology Criteria for Adverse Events v 5.0 throughout the study period and up to 30 days after the last dose. Toxicity was graded every week for the first five cycles and every 2 weeks thereafter, according to National Cancer Institute Common Terminology Criteria for Adverse Events v 5.0. Additional safety evaluations included physical examination, concomitant medications, cardiovascular assessment, vital signs and laboratory assessments which included blood count, clinical chemistry, including liver function test, coagulation and 12-lead electrocardiogram.

### Pharmacokinetics assessment

Serum tilvestamab levels were obtained from study participants on days 1, 2 and 8 during the first two cycles. Further serum samples were planned to be taken on days 1 and 8 of the third cycle and day 1 alone for the fourth and fifth cycles. For cycle 1 day 1, serum samples were collected at 0, 2, 4, 6, 8, 10, 24 h post-dose. Tumour biopsies were taken at screening and at cycle 2 day 2 of tilvestamab administration, timed to be close to the maximum serum exposure of tilvestamab. A schedule of the pharmacokinetic blood sampling schedule can be found in the supplementary index **(**supplementary material 2**)**.

Blood samples were centrifuged at 1500 g for ten minutes at 20 °C within 60 min of collection and serum stored at −80 °C within 120 min. Stored aliquots were shipped to a central laboratory in batches on dry ice for later analysis using validated ELISA method (Covance Laboratories Ltd., Harrogate, UK).

PK parameters (AUC0-168, Cmax, CL, Vss, terminal half-life) were derived from serum tilvestamab concentration versus time. The PK parameters for the first dose were calculated by a non-compartmental analysis using the computer software Phoenix™ WinNonlin® (Version 8.0, Certara Corporation). PK statistical analyses were performed by SAS® System Release (Version 9.4, SAS Institute Inc). Tissue from the tumour biopsies were used to characterise the tumour tissue PK in relation to overall plasma PK in simultaneous plasma samples.

### Immunogenicity assessments

Blood samples for the analysis of anti-drug antibodies (ADA) were taken for measurement of immunogenicity of tilvestamab.

### Efficacy and biomarker assessments

Treatment assessments included physical examination, serum CA125 and cross-sectional imaging with computed tomography (CT) scan of the thorax, abdomen and pelvis. Tumour assessment via scans were performed at screening and every 6 weeks during treatment, until disease progression. Measurable tumour responses were defined by Response Evaluation Criteria in Solid Tumours (RECIST), version 1.1. The disease control rate (DCR) was defined as the proportion of patients achieving complete response (CR), partial response (PR) or stable disease (SD) at 6 weeks. Biochemical responses were defined by Gynaecologic Cancer Intergroup (GCIG) criteria. PFS was calculated from the date of initiation of therapy to the first date when disease progression was documented by RECISTv1.1, or last follow-up. Patients who had disease progression required a confirmatory scan at least 4 weeks after the initial progression was first observed.

Patient-reported outcomes were measured via health-related quality of life (HRQoL) assessment using the European Organisation for Research and Treatment of Cancer (EORTC) QLQ-C30 (version 3) [38]. Patients would self-complete the questionnaire before any other study procedures took place at each designated visit. The questionnaire was administered at screening, every 6 weeks during treatment, and 30 days after treatment discontinuation.

### Pharmacodynamics assessments

Fresh tumour biopsies were mandated for all patients to be performed at baseline and at Cycle 2 Day 2 of the study. IHC studies were performed on tumour samples to determine AXL expression at baseline and after 4 weeks on treatment. Plasma and urine samples for PD analysis of tilvestamab were taken as per study schedule. The PD effects of tilvestamab in the blood and urine samples were to be determined using both validated and nonvalidated analytical methods.

DNA/RNA extraction was performed on up to 8 macrodissected sections per sample using a Qiagen AllPrep DNA/RNA FFPE (Formalin-Fixed Paraffin-Embedded) kit. RNA was processed using the Illumina TrueSeq RNA exome kit. Transcriptomics analyses were performed for GEMS profiling. Protein expression analyses were performed by reverse-phase protein arrays (RPPA) with 485 antibodies at the MD Anderson Functional Proteomics Core Facility [39].

For RNA-seq analysis and gene expression molecular subtype annotation, paired-end reads were aligned to hg38 genome using STAR v2.5.3a (Dobin 2013) and transcripts quantified using RSEM v1.3 (Li 2011) based on Gencode v30 annotation. The annotation of gene expression molecular subtypes was determined using a two-sample Kolmogorov-Smirnov method as outlined in the study by Tan et al. [40], along with previously defined subtype signatures [28]. Additional gene signatures tested include those related to fibrosis, inflammation, EMT, and NRF2.

### Statistical analysis

All patients who received at least one dose of tilvestamab were included in the safety, PK, immunogenicity and PD evaluations. All patients who received at least one dose of tilvestamab and had at least one postbaseline tumour scan were included in the evaluation of antitumor activity and response.

Median progression-free survival was estimated using the Kaplan-Meier method. Patient-reported HRQoL outcomes from the EORTC QLQ-C30 questionnaires were transformed linearly and analysed.

## Results

### Patient demographics

Between 25 February 2021 and 4 February 2022, a total of 16 patients were enroled from 8 sites in Singapore, Republic of Korea, United Kingdom, and Norway. There were 5 patients in cohort A, 6 patients in cohort B and 5 patients in cohort C. Median age was 56.0 years (range 51-84). 50% of patients were Asian, 6% patients were Pacific Islander and 44% were White in ethnicity. 50% of the patients had an ECOG performance status of 0 and 50% had performance status of 1. All patients had HGSOC histology. 81% had Stage 4 disease and 19% had Stage 3 disease at diagnosis. 25% of patients had 1 prior line of treatment, 31% had 2 prior lines of treatment and 44% of patients had at least 3 prior lines of treatment. 44% patients were positive for AXL IHC. The patient demographics can be seen in Table 1. Cessation of trial accrual at 16 out of the planned maximum 24 patients was primarily due to lack of efficacy signals and other strategic reasons.

**Table 1:** Patient demographics.

	Cohort A	Cohort B	Cohort C	Total
*N*	5	6	5	16
Age (median)	54	61	55	56
Ethnicity	Asian: 3 White: 2	Asian: 3 White: 2 Pacific Islander: 1	Asian: 2 White: 3	Asian: 8 White: 7 Pacific Islander: 1
Performance status	0: 3 1: 2	0: 3 1: 3	0: 2 1: 3	0: 8 1: 8
Weight (kg)	60.6	60.4	66.8	62.5
Height (cm)	155.9 (146.0-164.0)	156.7 (142.3-170.1)	167.2 (152.0-176.0)	159.7 (142.3-176.0)
HGSOC subtype	5	6	5	16
Stage	III: 1 IV: 4	III: 2 IV: 4	III: 0 IV: 5	III: 3 IV: 13
Previous lines of treatment	1 L: 0 2 L: 3 ≥3 L: 2	1 L: 2 2 L: 0 ≥3 L: 4	1 L: 2 2 L: 2 ≥3 L: 1	1 L: 4 2 L: 5 ≥3 L: 7
AXL expression at screening	Negative: 1 Positive: 2 Missing: 2	Negative: 1 Positive: 4 Missing: 1	Negative: 3 Positive: 1 Missing: 1	Negative: 5 Positive: 7 Missing: 4
Median treatment duration (weeks)	8.0 (3.9–17.6)	3.1 (0.1–10.0)	8.1 (4.1–21.1)	6.1 (0.1–21.1)
Median number of cycles completed	2.5 (1.5–4.5)	1.3 (0.5–3.5)	2.5 (1.5–5.5)	2.0 (0.5–5.5)
Median number of doses received	5 (3–9)	3 (1–6)	5 (3–11)	4 (1–11)

### Treatment duration

Of the 16 patients who entered the study, all (100.0%) discontinued due to disease progression. Among them, 2 patients (12.5%) were withdrawn based on clinical disease progression. The remaining 14 patients (87.5%) had disease progression seen on CT scans. However, only 2 of these patients (12.5%) underwent a repeat scan 28 days later, as required by protocol, to confirm progression prior to study discontinuation. For the other 12 patients (75.0%), the decision to omit the repeat confirmatory scan after a specified interval, were commensurate with the advanced nature of the disease under study and the need to proceed to alternative therapy without undue delay upon clinical progression. 5 of these patients (31.3%) fulfilled GCIG criteria for disease progression based on CA125.

Median number of tilvestamab cycles completed and total treatment duration on tilvestamab were 2.0 (0.5–5.5) and 6.1 (0.1–21.1) weeks, respectively. All but 1 patient in cohort B received all the doses as planned. All patients completed at least 1 cycle with tilvestamab, while only 1 patient in cohort C completed 5 cycles.

### Safety and tolerability

All 16 patients received at least 1 dose of tilvestamab and were evaluable for toxicity. 15 (94%) patients experienced a treatment-emergent AE and 10 (62.5%) patients experienced a grade 3 or higher AE. Across all grades, the most common adverse events were fatigue (38%), anorexia (38%) infections (31%), anaemia (25%) and dyspnoea (25%). As a group, high-grade toxicities were mainly gastrointestinal in nature. This included grade 3-4 intestinal obstruction (13%), abdominal pain (6%), abdominal distension (6%), nausea (6%), vomiting (6%). 1 (6%) patient experienced febrile neutropaenia on the study. Overall, there was no apparent correlation between dose and overall occurrence of adverse events, as patients in all cohorts reported events. Table 2A shows the breakdown of adverse events recorded.

**Table 2:** Adverse events.

Event	Cohort A (*n* = 5)		Cohort B (*n* = 6)		Cohort C (*n* = 5)		Total (*n* = 16)	
	Any grade	Grade ≥3	Any grade	Grade ≥3	Any grade	Grade ≥3	Any grade	Grade ≥3
**(A) Treatment-emergent adverse events (TEAE)**								
**Any**	5 (100)	4 (80)	5 (83)	3 (50)	5 (100)	3 (60)	15 (94)	10 (63)
**General**								
Fatigue	2 (40)	0 (0)	1 (17)	0 (0)	3 (60)	0 (0)	6 (38)	0 (0)
Anorexia	3 (60)	0 (0)	2 (33)	0 (0)	1 (20)	0 (0)	6 (38)	0 (0)
Dysgeusia	0 (0)	0 (0)	1 (17)	0 (0)	1 (20)	0 (0)	2 (13)	0 (0)
Oedema	0 (0)	0 (0)	1 (17)	0 (0)	2 (40)	0 (0)	3 (19)	0 (0)
Headache	1 (20)	0 (0)	0 (0)	0 (0)	1 (20)	0 (0)	2 (13)	0 (0)
Giddiness	1 (20)	0 (0)	0 (0)	0 (0)	1 (20)	0 (0)	2 (13)	0 (0)
Myalgia	0 (0)	0 (0)	1 (17)	0 (0)	1 (20)	0 (0)	2 (13)	0 (0)
**Gastrointestinal disorders**								
Abdominal pain	2 (40)	1 (20)	0 (0)	0 (0)	0 (0)	0 (0)	2 (13)	1 (6)
Abdominal distension	1 (20)	1 (20)	0 (0)	0 (0)	0 (0)	0 (0)	1 (6)	1 (6)
Nausea	2 (40)	1 (20)	0 (0)	0 (0)	1 (20)	0 (0)	3 (19)	1 (6)
Vomit	1 (20)	1 (20)	0 (0)	0 (0)	0 (0)	0 (0)	1 (6)	1 (6)
Constipation	2 (40)	0 (0)	1 (17)	0 (0)	0 (0)	0 (0)	3 (19)	0 (0)
Diarrhoea	2 (40)	0 (0)	0 (0)	0 (0)	0 (0)	0 (0)	2 (13)	0 (0)
Dry mouth	1 (20)	0 (0)	1 (17)	0 (0)	0 (0)	0 (0)	2 (13)	0 (0)
Intestinal obstruction	0 (0)	0 (0)	1 (17)	1 (17)	1 (20)	1 (20)	2 (13)	2 (13)
**Haematological abnormalities**								
Febrile neutropaenia	1 (20)	1 (20)	0 (0)	0 (0)	0 (0)	0 (0)	1 (6)	1 (6)
Neutropaenia	1 (20)	1 (20)	0 (0)	0 (0)	0 (0)	0 (0)	1 (6)	1 (6)
Thrombocytopaenia	1 (20)	1 (20)	0 (0)	0 (0)	0 (0)	0 (0)	1 (6)	1 (6)
Anaemia	2 (40)	1 (20)	0 (0)	0 (0)	2 (40)	0 (0)	4 (25)	1 (6)
**Laboratory abnormalities**								
Blood creatinine increase	1 (20)	0 (0)	2 (33)	1 (17)	0 (0)	0 (0)	3 (19)	1 (6)
Proteinuria	0 (0)	0 (0)	0 (0)	0 (0)	1 (20)	0 (0)	1 (6)	0 (0)
Alanine aminotransferase increase	1 (20)	1 (20)	0 (0)	0 (0)	0 (0)	0 (0)	1 (6)	1 (6)
Aspartate aminotransferase increase	1 (20)	1 (20)	0 (0)	0 (0)	0 (0)	0 (0)	1 (6)	1 (6)
Alkaline phosphatase increase	0 (0)	0 (0)	1 (17)	0 (0)	0 (0)	0 (0)	1 (6)	0 (0)
Hypoalbuminemia	0 (0)	0 (0)	1 (17)	0 (0)	1 (20)	0 (0)	2 (13)	0 (0)
Hypocalcaemia	1 (20)	0 (0)	0 (0)	0 (0)	0 (0)	0 (0)	1 (6)	0 (0)
Hypokalaemia	1 (20)	0 (0)	0 (0)	0 (0)	0 (0)	0 (0)	1 (6)	0 (0)
Hyponatremia	1 (20)	1 (20)	0 (0)	0 (0)	0 (0)	0 (0)	1 (6)	1 (6)
Hypophosphataemia	1 (20)	0 (0)	0 (0)	0 (0)	0 (0)	0 (0)	1 (6)	0 (0)
QTc prolongation	0 (0)	0 (0)	0 (0)	0 (0)	1 (20)	0 (0)	1 (6)	0 (0)
**Others**								
Hypertension	1 (20)	0 (0)	1 (17)	0 (0)	0 (0)	0 (0)	2 (13)	0 (0)
Venous thrombosis	0 (0)	0 (0)	0 (0)	0 (0)	1 (20)	1 (20)	1 (6)	1 (6)
Back pain	1 (20)	0 (0)	0 (0)	0 (0)	1 (20)	0 (0)	2 (13)	0 (0)
Dry skin	0 (0)	0 (0)	0 (0)	0 (0)	1 (20)	0 (0)	1 (6)	0 (0)
Rash	1 (20)	0 (0)	0 (0)	0 (0)	0 (0)	0 (0)	1 (6)	0 (0)
Dyspnoea	1 (20)	0 (0)	2 (33)	0 (0)	1 (20)	0 (0)	4 (25)	0 (0)
Insomnia	1 (20)	0 (0)	0 (0)	0 (0)	0 (0)	0 (0)	1 (6)	0 (0)
Infections	3 (60)	1 (20)	1 (17)	1 (17)	1 (20)	0 (0)	5 (31)	2 (13)
**(B) Treatment-related adverse events (TRAE) deemed related to tilvestamab**								
**Any**	5 (100)	0 (0)	2 (33)	0 (0)	3 (60)	0 (0)	10 (63)	0 (0)
**General**								
Fatigue	2 (40)	0 (0)	0 (0)	0 (0)	3 (60)	0 (0)	5 (31)	0 (0)
Anorexia	1 (20)	0 (0)	0 (0)	0 (0)	0 (0)	0 (0)	1 (6)	0 (0)
Dysgeusia	0 (0)	0 (0)	0 (0)	0 (0)	1 (20)	0 (0)	1 (6)	0 (0)
Oedema	0 (0)	0 (0)	0 (0)	0 (0)	2 (40)	0 (0)	2 (13)	0 (0)
Headache	1 (20)	0 (0)	0 (0)	0 (0)	1 (20)	0 (0)	2 (13)	0 (0)
Giddiness	0 (0)	0 (0)	0 (0)	0 (0)	0 (0)	0 (0)	0 (0)	0 (0)
Myalgia	0 (0)	0 (0)	1 (17)	0 (0)	1 (20)	0 (0)	2 (13)	0 (0)
**Gastrointestinal disorders**								
Abdominal pain	1 (20)	0 (0)	1 (17)	0 (0)	0 (0)	0 (0)	2 (13)	0 (0)
Vomit	0 (0)	0 (0)	1 (17)	0 (0)	0 (0)	0 (0)	1 (6)	0 (0)
Diarrhoea	1 (20)	0 (0)	0 (0)	0 (0)	0 (0)	0 (0)	1 (6)	0 (0)
Dry mouth	0 (0)	0 (0)	1 (17)	0 (0)	0 (0)	0 (0)	1 (6)	0 (0)
**Haematological abnormalities**								
Neutropaenia	1 (20)	0 (0)	0 (0)	0 (0)	0 (0)	0 (0)	1 (6)	0 (0)
Anaemia	1 (20)	0 (0)	0 (0)	0 (0)	1 (20)	0 (0)	2 (13)	0 (0)
**Laboratory abnormalities**								
QTc prolongation	0 (0)	0 (0)	0 (0)	0 (0)	1 (20)	0 (0)	1 (6)	0 (0)
**Others**								
Hypertension	0 (0)	0 (0)	1 (17)	0 (0)	0 (0)	0 (0)	1 (6)	0 (0)
Dry skin	0 (0)	0 (0)	0 (0)	0 (0)	1 (20)	0 (0)	1 (6)	0 (0)
Rash	1 (20)	0 (0)	0 (0)	0 (0)	0 (0)	0 (0)	1 (6)	0 (0)
Insomnia	1 (20)	0 (0)	0 (0)	0 (0)	0 (0)	0 (0)	1 (6)	0 (0)

When analysed by whether treatment was related to tilvestamab (Table 2B), the most common adverse events were fatigue (31%), oedema (13%), headache (13%), myalgia (13%), abdominal pain (13%) and anaemia (13%). There were no grade 3 adverse events deemed to be related to tilvestamab.

3 (18.8%) patients were discontinued from tilvestamab treatment due to adverse events, thought to be related to disease progression and unrelated to study drug, Specifically, 1 patient from cohort A was discontinued from treatment due to grade 3 abdominal pain considered to be unrelated to study drug. 1 patient from cohort B died due to disease progression. The last patient was from cohort C, where tilvestamab was discontinued due to skin oedema. This was attributed to disease progression, and thought to be unrelated to study drug.

### Pharmacokinetics

**Fig. 2:**
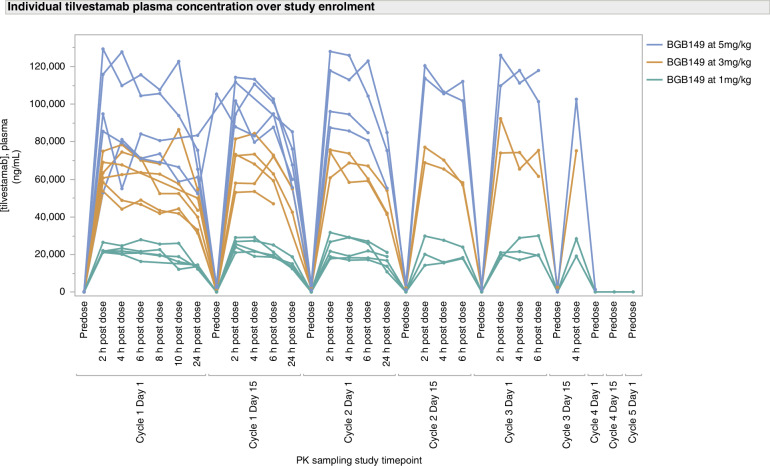
Tilvestamab concentration at various timepoints.

Tilvestamab exposure (Cmax and AUC) increased in a dose-proportional manner after single and multiple doses, with minimal accumulation. Steady state was reached after the second dose, with a mean t1/2 ranging from 47 to 69 h. Figure 2 shows the concentration of tilvestamab measured from each individual patient’s plasma sample at various time points. Serum PK parameters of tilvestamab by visit can be found in the supplementary index **(**supplementary material 3**)**.

### Immunogenicity

Blood samples from 12 patients were taken for immunogenicity analysis, Of the 12 patients, anti-drug antibodies (ADA) were detected in 2 patients, both from cohort A. The first patient had positive ADA prior to dosing at cycle 1 day 1; this was thought to be a false positive result as the patient had not had prior exposure to tilvestamab. Furthermore, the ADA testing results for this patient was inconsistent at subsequent timepoints. The second patient had a positive ADA result only during the follow-up visit. Overall, the detection of ADA at unexpected timepoints in these two patients raised doubts about the significance of the results, making them difficult to interpret meaningfully. As such, analysis of neutralising antibodies was not performed. ADA testing results for these 2 patients at various timepoints can be found in the supplementary index **(**supplementary material 4**)**.

### Efficacy and survival

Of the 16 patients treated on the study, all (100%) discontinued due to disease progression. 15 patients were eligible for radiological RECIST evaluation. There were no objective responses (PR or CR) observed across all cohorts. The best overall response was stable disease at 6 weeks in 7 (44%) patients, contributing to a 6-week DCR of 44%. Radiologic disease progression was seen in 8 (50%) patients at the first interval scan performed at 6 weeks. Table 3A shows the breakdown of ORR on the study.

**Table 3:** Response assessment at 6 weeks.

(A) All 16 patients				
	Cohort A (*N* = 5)	Cohort B (*N* = 6)	Cohort C (*N* = 5)	Total (*N* = 16)
Radiologic CR	0 (0)	0 (0)	0 (0)	0 (0)
Radiologic PR	0 (0)	0 (0)	0 (0)	0 (0)
Radiologic SD	3 (60.0)	1 (16.7)	3 (60.0)	7 (43.7)
	AXL + : 0	AXL + : 0	AXL + : 1	AXL + : 1
	AXL-: 1	AXL-: 1	AXL-: 1	AXL-: 3
	AXLu: 2	AXLu: 0	AXLu: 1	AXLu: 3
Radiologic PD	2 (40.0)	4 (66.7)	2 (40.0)	8 (50.0)
	AXL + : 2	AXL + : 3	AXL + : 0	AXL + : 5
	AXL-: 0	AXL-: 0	AXL-: 2	AXL-: 2
	AXLu: 0	AXLu: 1	AXLu: 0	AXLu: 1
Remark		1 subject (AXL + ) did not have CT after screening but met GCIG criteria for PD		

(B) All AXL+ patients				
	Cohort A (N = 2)	Cohort B (N = 4)	Cohort C (*N* = 1)	Total (*N* = 16)
Radiologic CR	0 (0)	0 (0)	0 (0)	0 (0)
Radiologic PR	0 (0)	0 (0)	0 (0)	0 (0)
Radiologic SD	0 (0)	0 (0)	1 (100.0)	1 (16.7)
Radiologic PD	2 (100.0)	3 (75.0)	0 (0)	5 (83.3)
Remark		1 subject (AXL + ) did not have CT after screening but met GCIG criteria for PD		

AXL + : Positive for AXL mutation.

AXL-: Negative for AXL mutation.

AXLu: Unknown AXL mutation status.

**Fig. 3:**
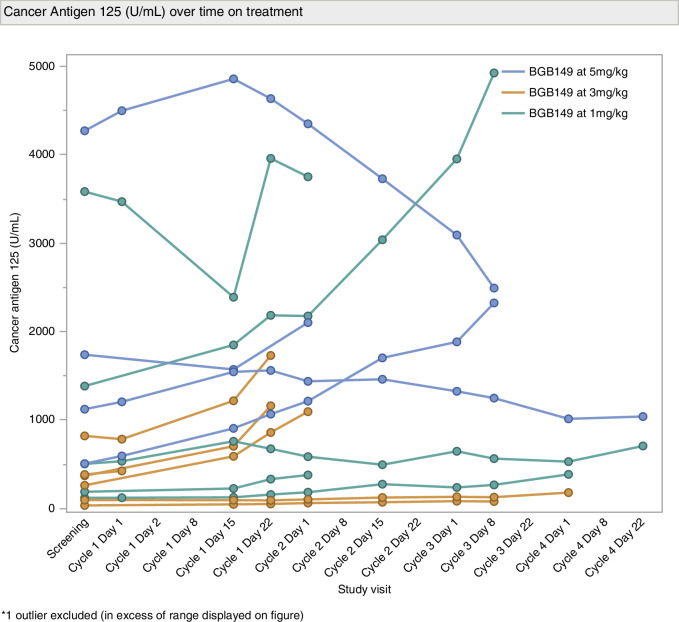
CA-125 trends.

**Fig. 4:**
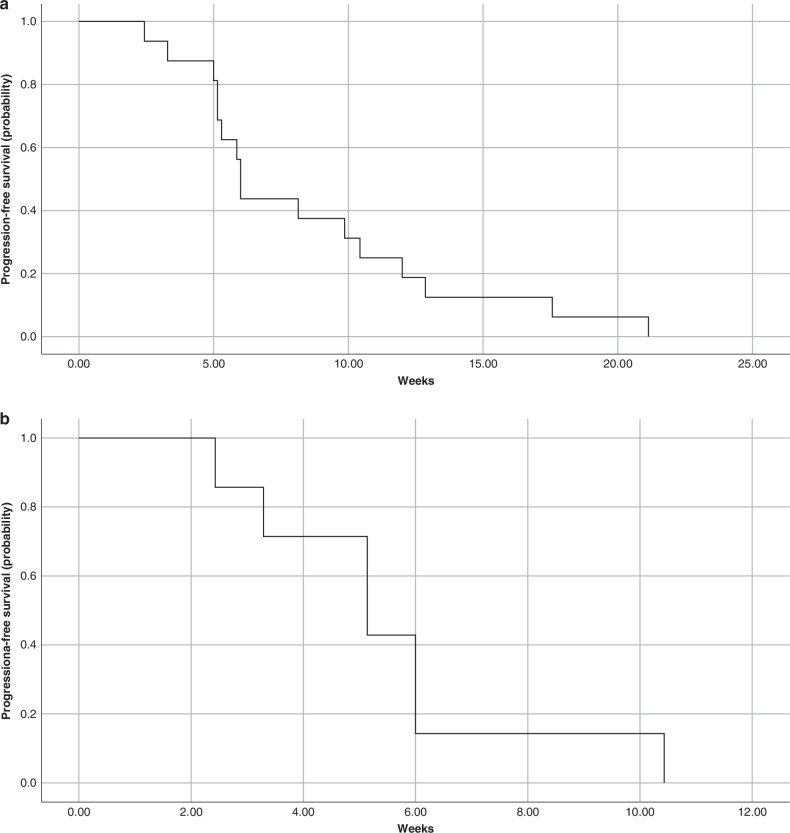
Progression-free survival. Progression-free survival curves by Kaplan-Meier method (**a** PFS in all 16 patients, **b** PFS in all AXL+ patients).

No patients exhibited a GCIG CA-125 response ( > 50% reduction from baseline), but 1 patient (AXL + ) from cohort C demonstrated a 48% reduction in CA-125 from peak level **(**Fig. 3**)**. The median PFS of all 16 patients was 6 weeks as shown in the Kaplan-Meier plot **(**Fig. 4a**)**.

Among the 16 patients, 7 were positive, 5 were negative and 4 were unknown for pre-treatment AXL expression status by IHC. When focusing solely on AXL expression positive patients, 6 out of 7 patients had radiological RECIST evaluation. The best overall response was stable disease in 1 (17%) patient, with 6-week DCR of 17%. The patient who did not perform CT scan for RECIST evaluation was taken off study on the basis of clinical disease progression, and her CA125 trend met disease progression criteria based on GCIG. The median PFS of all 7 AXL positive patients was 5 weeks **(**Fig. 4b**)**. A higher proportion of AXL negative (60%) patients had best overall response of radiological SD at 6 weeks compared to AXL positive (17%) patients. This suggests that AXL expression status by IHC did not correlate well with disease response on the study.

Baseline QOL assessments were completed by 100% (16/16) of patients. The global health status score changed from mean 68.2 at screening to 46.9 at follow-up but was not statistically significant. A similar observed downward trend in functional scales score was seen, however the change in scores were not statistically significant. With regards to symptom scales, a general increase in symptom burden over time was observed. In particular, the increment was significant for fatigue and nausea/vomiting during follow-up visit, as well as pain at the C2D15 visit. The change in other symptoms reported were not statistically significant. Of note, fewer patients participated in QOL assessments at later timepoints, largely due to disease progression. This may be commensurate with the aggressive nature of PROCs. QOL assessments at each scheduled timepoint can be found in the supplementary index **(**supplementary material 5**)**.

### Pharmacodynamics

**Fig. 5:**
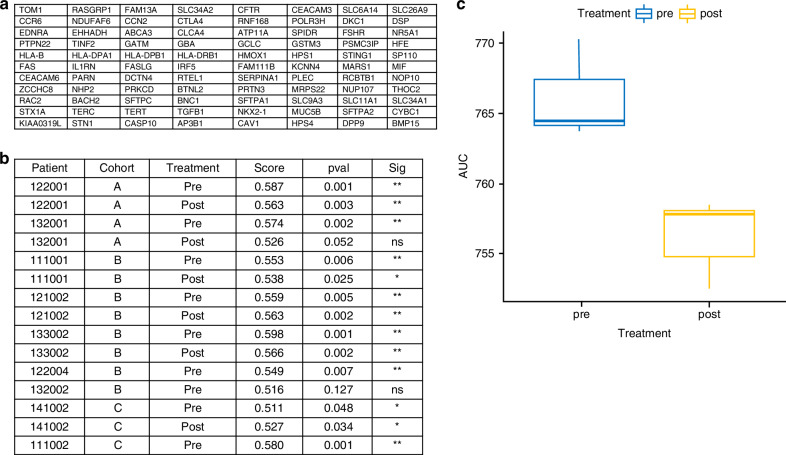
Fibrosis gene signatures analysis **a** Pulmonary fibrosis gene signature set that was tested, **b** 15 tumour specimens that underwent RNAseq analysis testing for the pulmonary fibrosis genes, **c** AUC comparison in fibrosis gene signature pre- and post-tilvestamab treatment.

Of 24 FFPE blocks received, 5 failed pathology quality control (inadequate tumour material present), and 4 failed RNA quality control. For the remaining 15 samples, 150–250 ng RNA was processed using the Illumina TrueSeq RNA exome kit, and subjected to 75 bp paired end read sequencing on the Illumina platform. An average of 42 M read pairs were obtained per sample. Signature enrichment between RNA sequencing data from biopsy samples pre- and post-treatment with tilvestamab were analysed. Gene signatures tested include those related to fibrosis, inflammation, EMT, and NRF2. Using the singscore method, it was found that the pulmonary fibrosis signature was significantly enriched in all but two samples **(**Fig. 5**)**. Comparison between pre- and post-treatment samples via AUC statistics showed a reduction in this fibrosis-related signature, confirming tilvestamab’s ability in modulating fibrogenic pathways. Further details regarding gene signatures tested are provided in the supplementary index **(**supplementary material 6**)**.

**Fig. 6:**
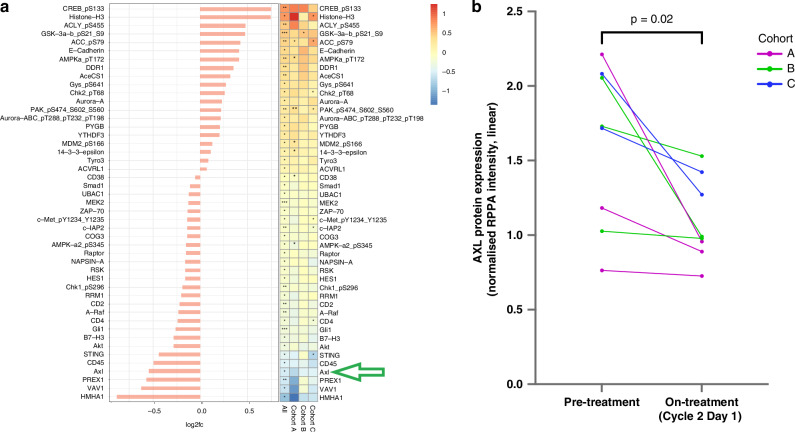
Reverse Phase Protein Arrays analysis. **a** Reverse Phase Protein Arrays in patients with paired samples showing changes in expression of multiple proteins, including downregulation of AXL expression, **b** AXL protein expression by RPPA.

**Fig. 7:**
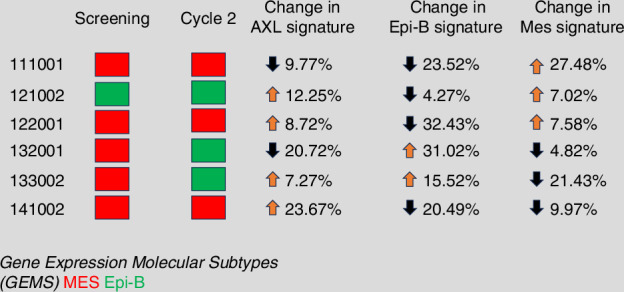
Gene expression molecular subtype (GEMS) profile of 6 patients with paired specimens by transcriptomics analysis.

Tissue biopsy samples and plasma were examined for protein biomarkers. 9 patients had paired samples available but 1 of them did not have sufficient protein for analysis. The remaining 8 paired samples (3 from cohort A, 3 from cohort B and 2 from cohort C) were analysed using RPPA, comparing baseline tissue specimen with that taken on cycle 2 day 2 of tilvestamab administration. RPPA analysis demonstrated in all samples that protein expression of AXL was consistently downregulated with treatment across all studied doses **(**Fig. 6a, b**)**. This is indicative of target engagement by tilvestamab. A reduction in AXL’s downstream target AKT (cellular homolog of murine thymoma virus akt8 oncogene) was seen, as evidenced by AMP-activated protein kinase (AMPK) activation [increase in E-cadherin and DDR1, increase in phosphorylated AKT substrates, including cAMP response element-binding protein (pCREB), glycogen synthase kinase 3 (pGSK3), and ATP citrate synthase (pACLY)] [41]. For GEMS profiling, 6 patients had paired samples available for analysis. Transcriptomics analysis showed that of the 6 patients, 5 had mesenchymal subtype PROCs. Treatment with tilvestamab caused a reduction in mesenchymal signature and increment in epithelial-B signature in 2 patients, essentially shifting the GEMS profile which may indicate reversal of EMT **(**Fig. 7**)**.

## Discussion

Tilvestamab is a highly selective full-length humanised immunoglobulin G1 anti-AXL antibody. AXL receptor tyrosine kinases are crucial in cancer cell proliferation, survival, metastasis, and drug resistance. These inhibitors are being tested in combination with other therapies, such as immune checkpoint inhibitors, tyrosine kinase inhibitors, and chemotherapy. They are particularly being examined in cancers where AXL overexpression is associated with poor prognosis and resistance to standard treatments, such as non-small cell lung cancer, triple-negative breast cancer, and acute myeloid leukaemia [42, 43].

This phase 1b, open-label, multicentre, multiple ascending dose study was conducted primarily to evaluate the safety and PK of tilvestamab given as monotherapy to adult patients with platinum-resistant HGSOC. Disease progression, efficacy based on RECIST v1.1 criteria, PFS and QOL parameters were explored in this study.

In this study, patients remained on treatment for median number of 2.0 (0.5–5.5) cycles and treatment duration of 6.1 (0.1–21.1) weeks, respectively. There were no objective responses and best response was SD achieved in 7 (44%) patients. One patient died during the study because of disease progression. A 6-week timepoint for response assessment is appropriate for PROCs because of poor response and progression-free survival in this group for patients, with patients typically having progression-free intervals of 8 weeks beyond 4 lines of systemic therapy after become platinum-resistant [44]. For PK measurements, tilvestamab exposure increased in a dose-proportional manner, steady state was reached after the second dose.

15 (94%) patients experienced an AE and 10 (62.5%) patients experienced a serious AE. AEs that lead to permanent discontinuation of tilvestamab were reported in 3 (18.8%) patients. High-grade toxicities were mainly gastrointestinal and haematological in nature, and 1 patient experienced febrile neutropaenia. There were no treatment-related serious AEs deemed related to tilvestamab. In terms of QOL measurements, there was no significant reduction in global health status or functional scales reported. Fatigue and nausea/vomiting were reported significantly by patients via QOL measurements. Of note, this may also be related to the underlying disease process, especially when considering lack of objective responses induced by treatment.

Whilst tilvestamab did not lead to objectively measurable responses irrespective of AXL expression status, our exploratory analyses of paired tumour biopsy specimens showed reduction in AXL protein expression, which led to AKT inhibition and AMPK activation. AMPK regulates autophagy and mitophagy through activation of the Unc-51-like kinase 1 (ULK1), the mammalian homologue of autophagy-related 1 (ATG1) [45]. This leads to tumour cells having an autophagic response to stress, shifting away from an EMT response. Such a phenomenon has been described by Marcucci et al. [46]. In our analysis, similar phenotypic changes in gene expression signature from mesenchymal to epithelial-B molecular subtype were observed in 2 patients. This was consistent with findings by Tan et al. [28], and provides pharmacodynamic proof of concept that AXL inhibition reverses the EMT transition in treatment-resistant HGSOC tumour cells. Furthermore, a reduction in pulmonary fibrosis signatures is suggestive of on-target effect of tilvestamab. This is relevant because the development of platinum resistance in ovarian cancer can be contributed by fibrosis resulting from prior chemotherapy [47] and cancer-associated fibroblasts [48].

Patients enroled on this study were not specifically selected to have mesenchymal subtype PROCs based on GEMS profiling. This could be a reason why no objective responses were seen. Additionally, the downregulation of AXL protein expression, reversal of EMT and reduction of pulmonary fibrosis signatures suggest on-target effect. This was however, contradicted by AXL IHC negative patients having a higher overall response than AXL IHC positive patients. Based on our findings, we postulate that tilvestamab alone may be insufficient to induce a response in PROCs. Rational next steps in clinical studies would be to investigate combination of tilvestamab with chemotherapy, given that reduction in the fibrotic stroma could allow for better penetration of chemotherapy into the tumour bed. As our findings suggest an absence of immunogenicity, tilvestamab and immunotherapy combinations may be less effective. Lastly, better selection of study population by looking at mesenchymal subtype PROCs is required.

This is also the only study that has looked at a pure AXL inhibitor in the form of a monoclonal antibody. Such a treatment strategy is less likely to lead to off-target effects and results from this study lends itself as a platform to study downstream consequences of AXL inhibition within the tumour and the microenvironment.

To conclude, tilvestamab administered every 14 days up to a maximum dose of 5 mg/kg was generally well-tolerated, for durations lasting up to 10 consecutive doses over 20 weeks. Pharmacokinetics were consistent with that previously observed in the first-time-in-human study [49]. In this study, dose proportional exposure was demonstrated with achievement of steady-state by the second dose and minimal accumulation over time. This study suggests that AXL inhibition with tilvestamab does not lead to measurable anti-tumour responses in patients with PROC. Whether this is true for other disease areas should be the focus of follow-on studies. Encouraging data on the switch in gene expression subtype on longitudinal samples obtained from patients following exposure to tilvestamab suggests on-target inhibitor of the AXL pathway with associated phenotypic changes in gene expression subtype, and further studies are required.

## Supplementary information

Supplementary index

## Data Availability

The data that supports the findings of this study are available from BerGenBio but restrictions apply to the availability of these data, which were used under license for the current study, and so are not publicly available. Data are however available from the authors upon reasonable request and with permission of BerGenBio.
