# Supplementary material for: A phase 1b, multicentre, dose escalation, safety and pharmacokinetics study of tilvestamab (BGB149) in relapsed, platinum-resistant, high-grade serous ovarian cancer (PROC) patients
Source: Br J Cancer. 2025 Jul 22;133(6):896–908. doi: 10.1038/s41416-025-03090-6 (PMC12449448; doi:10.1038/s41416-025-03090-6)
Supplement: Supplementary file 1 — Supplementary index [file 41416_2025_3090_MOESM1_ESM.docx]

Supplementary material 1: Findings from a single ascending dose study of tilvestamab in healthy male volunteers (unpublished)

A phase 1b single ascending dose study of tilvestamab (BGB149-101, EudraCT 2018-003186-33) was conducted in 24 healthy male volunteers, receiving a single ascending dose between 0.1mg/kg to 3.0 mg/kg or placebo. In this study, 42 adverse events were reported, with 13 related to treatment of which 5 linked to active treatment with tilvestamab (lower abdominal pain, back pain, myalgia, dizziness and headache).

Most events were mild, with one serious adverse event of a workplace accident (unrelated to the study or treatment), with no fatalities or discontinuations occurring. Laboratory values, vital signs, ECG measurements, and physical exams showed no significant changes, and all blood samples tested negative for anti-drug antibodies.

Supplementary material 2: Pharmacokinetic blood sampling schedule


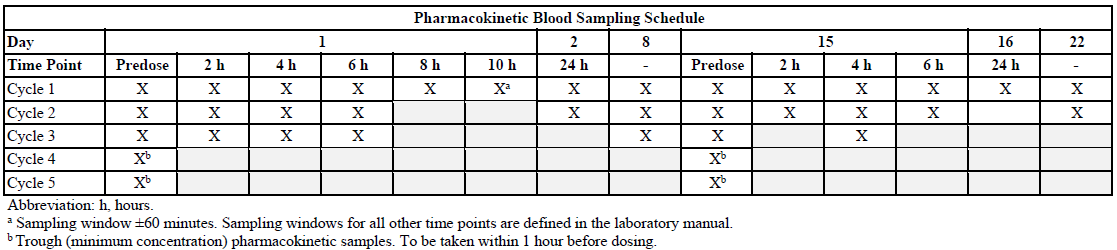


Supplementary material 3: Serum PK parameters of Tilvestamab by visit

| Visit | Parameter | Statistic | Cohort A  1mg/kg  (N=5) | Cohort B  3mg/kg  (N=6) | Cohort C  5mg/kg  (N=5) |
| --- | --- | --- | --- | --- | --- |
| C1D1 | C_max_ (ng/mL) | n | 5 | 6 | 5 |
|  |  | Mean (SD) | 23145 (2687) | 67466 (11807) | 103527 (23194) |
|  | AUC_inf_ (h×ng/mL) | n | 5 | 6 | 5 |
|  |  | Mean (SD) | 1367072 (189242) | 5095777 (1160557) | 7391959 (1901142) |
|  | AUC_last_ (h×ng/mL) | n | 5 | 6 | 5 |
|  |  | Mean (SD) | 1213806 (137278) | 4771606 (1189865) | 6908066 (1652874) |
|  | AUC_0-tau_ (h×ng/mL) | n | 5 | 6 | 5 |
|  |  | Mean (SD) | 1389461 (179302) | 4959589 (1070918) | 7182901 (1762659) |
|  | t_1/2_ (h) | n | 5 | 6 | 5 |
|  |  | Mean (SD) | 48.84 (5.993) | 63.90 (7.055) | 60.26 (14.137) |
| C1D15 | C_max_ (ng/mL) | n | 5 | 5 | 5 |
|  |  | Mean (SD) | 25381 (2919) | 71169 (11139) | 106438 (8095) |
|  | AUC_las_t (h×ng/mL) | n | 5 | 5 | 5 |
|  |  | Mean (SD) | 1504828 (472991) | 5246919 (1303015) | 7492010 (1670081) |
|  | AUC_0-tau_ (h×ng/mL) | n | 5 | 5 | 5 |
|  |  | Mean (SD) | 1829942 (751820) | 5863702 (1167351) | 8300169 (993492) |
|  | t_1/2_ (h) | n | 5 | 5 | 5 |
|  |  | Mean (SD) | 68.25 (25.012) | 65.87 (0.551) | 63.43 (10.385) |
| C2D1 | C_max_ (ng/mL) | n | 5 | 3 | 4 |
|  |  | Mean (SD) | 23845 (6087) | 72802 (3741) | 108422 (19884) |
|  | AUC_las_t (h×ng/mL) | n | 5 | 3 | 4 |
|  |  | Mean (SD) | 1597548 (492747) | 4938528 (1059790) | 6805860 (3500317) |
|  | AUC_0-tau_ (h×ng/mL) | n | 5 | 3 | 4 |
|  |  | Mean (SD) | 1853227 (838039) | 5082541 (860500) | 7841556 (2401600) |
|  | t_1/2_ (h) | n | 5 | 3 | 4 |
|  |  | Mean (SD) | 61.08 (18.182) | 61.97 (9.567) | 55.85 (18.450) |
| C2D15 | C_max_ (ng/mL) | n | 3 | 2 | 2 |
|  |  | Mean (SD) | 22466 (6341) | 72798 (5819) | 116896 (4670) |
|  | AUC_las_t (h×ng/mL) | n | 3 | 2 | 2 |
|  |  | Mean (SD) | 1537854 (187347) | 5307152 (3112) | 9328958 (623497) |
|  | AUC_0-tau_ (h×ng/mL) | n | 3 | 2 | 2 |
|  |  | Mean (SD) | 1687060 (270111) | 5258021 (68838) | 9300177 (581450) |
|  | t_1/2_ (h) | n | 3 | 2 | 2 |
|  |  | Mean (SD) | 52.45 (4.738) | 69.30 (5.091) | 56.90 (8.910) |
| C3D1 | C_max_ (ng/mL) | n | 3 | 2 | 2 |
|  |  | Mean (SD) | 23756 (5317) | 83094 (12720) | 121734 (5692) |
|  | AUC_las_t (h×ng/mL) | n | 3 | 2 | 2 |
|  |  | Mean (SD) | 1492237 (290871) | 5494876 (1083998) | 7342130 (341194) |
|  | AUC_0-tau_ (h×ng/mL) | n | 3 | 2 | 2 |
|  |  | Mean (SD) | 1712896 (NA) | 6350373 (NA) | 8499079 (NA) |
|  | t_1/2_ (h) | n | 3 | 2 | 2 |
|  |  | Mean (SD) | 55.20 (NA) | 57.20 (NA) | 47.00 (NA) |

Supplementary material 4: ADA testing results of 2 patients

| ADA | Patient 1 | Patient 2 |
| --- | --- | --- |
| Cycle 1, day 1 | Positive | Negative |
| Cycle 2, day 1 | Negative | Negative |
| Cycle 3, day 1 | Positive | Negative |
| Follow-up | Negative | Positive |

Supplementary material 5: Quality of life assessment

| Scale | Timepoint | N | Mean | SD | t-test (compared to screening) |
| --- | --- | --- | --- | --- | --- |
| **Global health status/QoL** | | | | | |
| Global health status | Screening | 16 | 68.2 | 25.3 | NA |
| (Score range 0-14) | C1D1 | 16 | 65.1 | 20.5 | M= 3.1 SD= 16.6. t(16)= 0.75, p= 0.464 |
|  | C2D15 | 8 | 68.8 | 25.5 | M= 9.4 SD= 19.1. t(8)= 1.39, p= 0.208 |
|  | C4D1 | 4 | 60.4 | 31.5 | M= 18.8 SD= 17.2. t(4)= 2.18, p= 0.117 |
|  | Follow-up | 8 | 46.9 | 32.1 | M= 17.7 SD= 26.1. t(8)= 1.92, p= 0.096 |
| **Functional scales** | | | | | |
| Physical functioning | Screening | 16 | 77.5 | 15.6 | NA |
| (Score range 0-20) | C1D1 | 16 | 77.9 | 14.5 | M= -0.4 SD= 9.3. t(16)= -0.18, p= 0.863 |
|  | C2D15 | 8 | 67.5 | 26.8 | M= 16.7 SD= 24.9. t(8)= 1.89, p= 0.101 |
|  | C4D1 | 4 | 70.0 | 15.9 | M= 16.7 SD= 20.7. t(4)= 1.61, p= 0.206 |
|  | Follow-up | 8 | 54.2 | 35.7 | M= 22.5, SD= 27.6. t(8)= 2.31, p= 0.054 |
| Role functioning | Screening | 16 | 68.8 | 30.3 | NA |
| (Score range 0-8) | C1D1 | 16 | 65.6 | 33.0 | M= 3.1 SD= 16.4. t(16)= 0.77, p= 0.455 |
|  | C2D15 | 8 | 60.4 | 39.8 | M= 22.9 SD= 13.0. t(8)= 1.77, p= 0.120 |
|  | C4D1 | 4 | 54.2 | 37.0 | M= 29.2 SD= 31.8. t(4)= 1.48, p= 0.235 |
|  | Follow-up | 8 | 45.8 | 35.4 | M= 20.8 SD= 31.8. t(8)= 1.85, p= 0.106 |
| Emotional functioning | Screening | 16 | 79.2 | 20.6 | NA |
| (Score range 0-16) | C1D1 | 16 | 82.8 | 18.9 | M= -3.6 SD= 12.9. t(16)= -1.13, p= 0.277 |
|  | C2D15 | 8 | 69.7 | 38.6 | M= 6.3 SD= 18.2. t(8)= 0.97, p= 0.364 |
|  | C4D1 | 4 | 58.3 | 44.1 | M= 6.3 SD= 36.2. t(4)= 0.35, p= 0.753 |
|  | Follow-up | 8 | 65.6 | 34.3 | M= 12.5 SD= 23.6. t(8)= 1.50, p= 0.177 |
| Cognitive functioning | Screening | 16 | 72.9 | 29.7 | NA |
| (Score range 0-8) | C1D1 | 16 | 76.0 | 32.2 | M= -3.1 SD= 13.9. t(16)= -0.90, p= 0.382 |
|  | C2D15 | 8 | 66.7 | 34.5 | M= -4.2 SD= 11.8. t(8)= -1.01, p= 0.348 |
|  | C4D1 | 4 | 62.5 | 28.5 | M= -12.5 SD= 28.4. t(4)= -0.88, p= 0.444 |
|  | Follow-up | 8 | 62.5 | 44.3 | M= 2.1, SD= 16.5. t(8)= -0.36, p= 0.733 |
| Social functioning | Screening | 16 | 68.7 | 25.7 | NA |
| (Score range 0-8) | C1D1 | 16 | 74.0 | 19.2 | M= -5.2 SD= 23.4. t(16)= -0.89, p= 0.385 |
|  | C2D15 | 8 | 66.7 | 36.7 | M= 6.2 SD= 19.8. t(8)= 0.89, p= 0.402 |
|  | C4D1 | 4 | 66.7 | 27.2 | M= -4.2 SD= 21.0. t(4)= -0.40, p= 0.716 |
|  | Follow-up | 8 | 47.9 | 37.2 | M= 18.8 SD= 31.4. t(8)= 1.69, p= 0.135 |
| **Symptoms scale/items** | | | | | |
| Fatigue | Screening | 16 | 33.3 | 21.1 | NA |
| (Score range 0-12) | C1D1 | 16 | 34.0 | 23.8 | M= -0.7 SD= 17.0. t(16)=-0.17, p= 0.871 |
|  | C2D15 | 8 | 38.9 | 37.6 | M= -0.7 SD= 20.5. t(8)= -0.96, p= 0.370 |
|  | C4D1 | 4 | 44.5 | 37.4 | M= -2.8 SD= 29.2. t(4)= -0.19, p= 0.862 |
|  | Follow-up | 8 | 56.9 | 36.4 | M= -18.1 SD= 21.4. t(8)= -2.39, p= 0.048 |
| Nausea and vomiting | Screening | 16 | 14.1 | 20.1 | NA |
| (Score range 0-8) | C1D1 | 16 | 11.5 | 18.0 | M= 2.7 SD= 19.2. t(16)= 0.55, p= 0.589 |
|  | C2D15 | 8 | 14.6 | 24.3 | M= -1.0 SD= 24.6. t(8)= -0.11, p= 0.916 |
|  | C4D1 | 4 | 33.3 | 47.1 | M= -26.9 SD= 39.3. t(4)= -1.37, p= 0.265 |
|  | Follow-up | 8 | 33.3 | 29.5 | M= -23.9 SD= 26.6. t(8)= -2.54, p= 0.039 |
| Pain | Screening | 16 | 32.3 | 29.5 | NA |
| (Score range 0-8) | C1D1 | 16 | 27.1 | 25.7 | M= 5.2 SD= 11.7. t(16)= 1.78, p= 0.096 |
|  | C2D15 | 8 | 41.7 | 43.6 | M= -12.5 SD= 14.8. t(8)= -2.39, p= 0.048 |
|  | C4D1 | 4 | 37.6 | 36.9 | M= 8.3 SD= 16.7. t(4)= 1.00, p= 0.391 |
|  | Follow-up | 8 | 50.0 | 41.8 | M= -16.7 SD= 21.8. t(8)= -2.16, p= 0.068 |
| Dyspnoea | Screening | 16 | 31.2 | 31.0 | NA |
| (Score range 0-4) | C1D1 | 16 | 27.1 | 25.0 | M= 4.2 SD= 29.5. t(16)= 0.57, p= 0.581 |
|  | C2D15 | 8 | 37.5 | 37.5 | M= -16.7 SD= 35.6. t(8)= -1.32, p= 0.227 |
|  | C4D1 | 4 | 25.0 | 31.9 | M= -16.7 SD= 33.4. t(4)= -1.00, p= 0.391 |
|  | Follow-up | 8 | 41.7 | 29.6 | M= -20.8 SD= 39.6. t(8)= -1.49, p= 0.181 |
| Insomnia | Screening | 16 | 31.2 | 28.5 | NA |
| (Score range 0-4) | C1D1 | 16 | 27.1 | 30.4 | M= 4.2 SD= 20.6. t(16)= 0.81, p= 0.432 |
|  | C2D15 | 8 | 33.3 | 35.6 | M= 8.3 SD= 34.5. t(8)= 0.68, p= 0.517 |
|  | C4D1 | 4 | 41.7 | 41.9 | M= 16.7 SD= 19.3. t(4)= 1.73, p= 0.182 |
|  | Follow-up | 8 | 54.2 | 35.4 | M= -20.8 SD= 30.5. t(8)= -1.93, p= 0.095 |
| Appetite loss | Screening | 16 | 20.8 | 26.9 | NA |
| (Score range 0-4) | C1D1 | 16 | 16.7 | 21.1 | M= 4.2 SD= 20.6. t(16)= 0.81, p= 0.432 |
|  | C2D15 | 8 | 33.3 | 39.8 | M= -20.9 SD= 35.4. t(8)= -1.67, p= 0.139 |
|  | C4D1 | 4 | 41.7 | 50.0 | M= -25.0 SD= 68.7. t(4)= -0.73, p= 0.519 |
|  | Follow-up | 8 | 54.2 | 43.4 | M= -29.2 SD= 41.5, t(8)= -1.99, p= 0.087 |
| Constipation | Screening | 16 | 25.0 | 33.3 | NA |
| (Score range 0-4) | C1D1 | 16 | 18.7 | 29.7 | M= 6.2 SD= 18.1. t(16)= 1.38, p= 0.188 |
|  | C2D15 | 8 | 33.3 | 43.6 | M= -12.5 SD= 39.6. t(8)= -0.89, p= 0.401 |
|  | C4D1 | 4 | 41.7 | 50.0 | M= -16.7 SD= 57.7. t(4)= -0.56, p= 0.604 |
|  | Follow-up | 8 | 45.8 | 30.6 | M= -12.5 SD= 50.2. t(8)= -0.70, p= 0.504 |
| Diarrhoea | Screening | 16 | 8.3 | 14.9 | NA |
| (Score range 0-4) | C1D1 | 16 | 6.2 | 13.4 | M= 2.1 SD= 8.3. t(16)= 1.01, p= 0.329 |
|  | C2D15 | 8 | 4.2 | 11.8 | M= 8.3 SD= 15.4. t(8)= 1.53, p= 0.170 |
|  | C4D1 | 4 | 8.3 | 16.7 | M= 8.3 SD= 31.9. t(4)= 0.52, p= 0.638 |
|  | Follow-up | 8 | 12.5 | 24.8 | M= -0.01 SD= 17.8. t(8)= -0.002, p= 0.998 |
| Financial difficulties | Screening | 16 | 14.6 | 21.0 | NA |
| (Score range 0-4) | C1D1 | 16 | 10.4 | 20.1 | M= 4.2 SD= 11.4. t(16)= 1.46, p= 0.164 |
|  | C2D15 | 8 | 16.7 | 25.2 | M= -4.2 SD= 11.8. t(8)= -1.00, p= 0.351 |
|  | C4D1 | 4 | 25.0 | 31.9 | M= -16.7 SD= 33.4. t(4)= -1.00, p= 0.391 |
|  | Follow-up | 8 | 12.5 | 17.2 | t-test cannot be computed because standard error of the difference is 0 |

Supplementary material 6: Gene signatures tested

We initially looked five fibrotic signatures found in molsigdb in view of interest in role of AXL in fibrosis, as listed:

- RENAL_FIBROSIS
- LUNG_FIBROSIS
- PULMONARY_FIBROSIS
- HEPATIC_FIBROSIS
- PANCREATIC_FIBROSIS

**Of these, only the Pulmonary Fibrosis gene signature was positive at p=0.007.**

We subsequently tested the following, without detecting any significance in any of them:

Inflammation-related

- GOBP_ACUTE_INFLAMMATORY_RESPONSE
- GOBP_INFLAMMATORY_RESPONSE
- HALLMARK_INFLAMMATORY_RESPONSE
- WP_RESOLVIN_E1_AND_RESOLVIN_D1_SIGNALING_PATHWAYS_PROMOTING_INFLAMMATION_RESOLUTION

EMT-related

- REACTOME_TGF_BETA_RECEPTOR_SIGNALING_IN_EMT
- METASTASIS_EMT
- FOROUTAN_INTEGRATED_TGFB_EMT
- FOROUTAN_TGFB_EMT

NRF2 (NFE2L2)-related

- NFE2L2.V2
- REACTOME_KEAP1_NFE2L2_PATHWAY
- REACTOME_NUCLEAR_EVENTS_MEDIATED_BY_NFE2L2
- SINGH_NFE2L2_TARGETS
- BIOCARTA_ARENRF2_PATHWAY
- WP_NRF2_PATHWAY
- WP_NRF2ARE_REGULATION
- IBRAHIM_NRF2
